# Variability of oxygen requirements in critically ill COVID-19 patients

**DOI:** 10.7189/jogh.14.05012

**Published:** 2024-02-23

**Authors:** Samuel F Huth, Alexander Rothkopf, Lisa Smith, Nicole White, Gianluigi Li Bassi, Jacky Y Suen, John F Fraser, COVID-19 Critical Care Consortium, John Laffey, Daniel Brodie, Eddy Fan, Antoni Torres, Davide Chiumello, Alyaa Elhazmi, Carol Hodgson, Shingo Ichiba, Carlos Luna, Srinivas Murthy, Alistair Nichol, Pauline Yeung Ng, Mark Ogino, Eva Marwali, Giacomo Grasselli, Robert Bartlett, Aidan Burrell, Muhammed Elhadi, Anna Motos, Ferran Barb, Alberto Zanella, Tala Al-Dabbous, Huda Alfoudri, Mohammed Shamsah, Khadeejeh Alfroukh, Zinah Aqeel Abdulzahra Bairmani, Khalid Jehad Khalid, Salsabeel MA Abukhalaf, Mohammed Maher Hadhoud, Mohamed Fathi, Hasan Alhouri, Hamza Shahla, Qamrah Alhadad, Matly Hanan, Subbarao Elapavaluru, Ashley Berg, Christina Horn, Ahmed Reda Mohamed Elsayed Abdelhalim, Amro Essam Amer, Cinderella Omar Rageh Elnaggar, Ahmed Ayman Hassan, Ali Abdelaziz, Mohamed Abdelhalim, Yehia Samir Shaaban Aly Orabi, Zinah A Alaraji, Mo'nes R Muhaisen, Lana Almasri, Dana Mustafa, Shaher Hamdan, Yousef Al-Saba'a, Zaina Dalloul, Mohammed Alkahlout, Hamza Jaber, Osama Aldabbourosama, Alaa Abdalfattah Abdalhadi, Aliae AR Mohamed Hussein, Zarief Kamel Emad, Sarah Khaled, Nouralsabah Mohamed, Ebtisam Hassanin, Abdelhafeez Hamdi, May Gamal, Ahmed Emad, Abdelrahman Ragab, Mohammed G Azizeldin, Almthani Hamza, Alsarrah Ali Mohammed Omer, Asgad Osman Abdalla Fadl alla, Asia Atif Abdelrahman Abdallahrs, Aml Ahmed Eltayeb, Maali khalid mohamed abdalla Alhasan, Esraa Hassan Abdelgaum, Aya Mustafa Ahmed, Lamees Adil Abdulbaqi, Omer Abdullah Mohammedelhassan, Musaab Mohammed Mohammed Ahmed, Maha TagElser Mohammed Ali, Yunis Mayasi, Stephan Schroll, Dan Meyer, Jorge Velazco, Ludmyla Ploskanych, Wanda Fikes, Rohini Bagewadi, Marvin Dao, Haley White, Alondra Berrios Laviena, Ashley Ehlers, Maysoon Shalabi-McGuire, Trent Witt, Lorenzo Grazioli, Luca Lorini, E Wilson Grandin, Jose Nunez, Tiago Reyes, Diarmuid O'Briain, Stephanie Hunter, Mahesh Ramanan, Julia Affleck, Hemanth Hurkadli Veerendra, Sumeet Rai, Josie Russell-Brown, Mary Nourse, Mark Joseph, Brook Mitchell, Martha Tenzer, Ryuzo Abe, Hwa Jin Cho, In Seok Jeong, Nadeem Rahman, Vivek Kakar, Ahmed Tamimi, Diala Zabalawi, Mohamed Elhennawi, Praveen Ghisulal, Sadaf Malik, Nicolas Brozzi, Omar Mehkri, Sudhir Krishnan, Abhijit Duggal, Stuart Houltham, Jeronimo Graf, Roderigo Diaz, Roderigo Orrego, Camila Delgado, Joyce González, Maria Soledad Sanchez, Michael Piagnerelli, Josefa Valenzuela Sarrazin, Gustavo Zabert, Lucio Espinosa, Paulo Delgado, Victoria Delgado, Diego Fernando Bautista Rincón, Angela Maria Marulanda Yanten, Melissa Bustamante Duque, Daniel Brodie, Khaled Abouelmagd, Alyaa Elhazmi, Abdullah Al-Hudaib, Jeff Javidfar, Maria Callahan, Andy Dong, Charles Crepy D'Orleans, M Azhari Taufik, Elizabeth Yasmin Wardoyo, Margaretha Gunawan, Nurindah S Trisnaningrum, Vera Irawany, Muhammad Rayhan, Mauro Panigada, Antonio Pesenti, Alberto Zanella, Giacomo Grasselli, Sebastiano Colombo, Chiara Martinet, Gaetano Florio, Massimo Antonelli, Simone Carelli, Domenico L Grieco, Motohiro Asaki, Kota Hoshino, Leonardo Salazar, Mary Alejandra Mendoza Monsalve, John Laffey, Bairbre McNicholas, David Cosgrave, Minha Atif, Fadi Qutishat, Caoimhe Laffey, Michaeal Van Der Walt, Joseph McCaffrey, Allison Bone, Jemma Trickey, Michelle Horton, Michelle Horton, Stephanie Pearce, Tania Salerno, Akram Mohamed, Salem Alhaddad, Baliad Bakeer, Shames Haitam, Laila Shalabi, Mohammed Abodina Ahmed, Yusuff Hakeem, James Winearls, Mandy Tallott, David Thomson, Ivan Joubert, Christel Arnold-Day, Jenna Piercy, Richard van Zyl Smit, Malcom Miller, Lisa Seymour, Francois van Heyningen, Gilbert Teyangesikayi, David Fredericks, Ali Ait Hssain, Jeffrey Aliudin, Al-Reem Alqahtani, Khoulod Mohamed, Ahmed Mohamed, Darwin Tan, Joy Villanueva, Ahmed Zaqout, Ahmed Labib, Ethan Kurtzman, Arben Ademi, Ana Dobrita, Khadija El Aoudi, Juliet Segura, Gezy Giwangkancana, Shinichiro Ohshimo, Javier Osatnik, Anne Joosten, Antoni Torres, Minlan Yang, Ana Motos, Carlos Luna, Francisco Arancibia, Virginie Williams, Alexandre Noel, Nestor Luque, Marina Fantini, Ruth Noemi Jorge Garcia, Enrique Chicote Alvarez, Anna Greti, Adrian Ceccato, Angel Sanchez, Ana Loza Vazquez, Ferran Roche-Campo, Diego Franch-Llasat, Divina Tuazon, Marcelo Amato, Luciana Cassimiro, Flavio Pola, Francis Ribeiro, Guilherme Fonseca, Heidi Dalton, Mehul Desai, Erik Osborn, Hala Deeb, Antonio Arcadipane, Gennaro Martucci, Giovanna Panarello, Stefano Vitiello, Claudia Bianco, Giovanna Occhipinti, Matteo Rossetti, Raffaele Cuffaro, Nidhal Siddig, Sung-Min Cho, Glenn Whitman, Marwan El Sayed, Walaa Mokhtar, Eslam El-Shenawy, Hiroaki Shimizu, Naoki Moriyama, Jae-Burm Kim, Nobuya Kitamura, Johannes Gebauer, Toshiki Yokoyama, Abdulrahman Al-Fares, Sarah Buabbas, Esam Alamad, Fatma Alawadhi, Kalthoum Alawadi, Mohamed Ahmed Khalefa, Nourah Ahmad Abdulaziz Al Ajeel, Mohammad Fathy Aly, Abdullah Al-Saleh, Abdullah Naanouh, Alaa Mohammed Elshourbgy, Abdulrahman Al-Fares, Mohamed Yousef Gad, Rania Mohamed ElRazaz, Ibrahim Khadadah, Ahmed Mohammed Almumin, Hala Altarakma, Hasan Albannay, Mohammed Kh Alsaleh, Mahmoud Saad Abdallah Radwan, Islam Ahmed Saadallah, Hiro Tanaka, Satoru Hashimoto, Masaki Yamazaki, Tak-Hyuck Oh, Mark Epler, Cathleen Forney, Louise Kruse, Jared Feister, Joelle Williamson, Katherine Grobengieser, Eric Gnall, Sasha Golden, Mara Caroline, Timothy Shapiro, Colleen Karaj, Lisa Thome, Lynn Sher, Mark Vanderland, Mary Welch, Sherry McDermott, Matthew Brain, Sarah Mineall, Maria Unwin, Lixian Chen, Tarnya Trezise, Laurie McKeon, Dai Kimura, Luca Brazzi, Gabriele Sales, Giorgia Montrucchio, Tawnya Ogston, Dave Nagpal, Karlee Fischer, Roberto Lorusso, Bas van Bussell, Maria Elena De Piero, Silvia Mariani, Rajavardhan Rangappa, Rajesh Mohan Shetty, Sujin Rai P, Argin Ganesan, Samar Tharwat, Mariano Esperatti, Nora Angélica Fuentes, Maria Eugenia Gonzalez, Diarmuid O'Briain, Edmund G Carton, Ayan Sen, Amanda Palacios, Deborah Rainey, Gordan Samoukoviv, Josie Campisi, Lucia Durham, Emily Neumann, Cassandra Seefeldt, Octavio Falcucci, Amanda Emmrich, Jennifer Guy, Carling Johns, Kelly Potzner, Catherine Zimmermann, Angelia Espinal, Nina Buchtele, Michael Schwameis, Andrea Korhnfehl, Roman Brock, Thomas Staudinger, Stephanie-Susanne Stecher, Michaela Barnikel, Sofia Anton, Alexandra Pawlikowski, Akram Zaaqoq, Lan Anh Galloway, Caitlin Merley, Mohamed Muftah, Alistair Nichol, Marc Csete, Luisa Quesada, Isabela Saba, Daisuke Kasugai, Hiroaki Hiraiwa, Taku Tanaka, Eva Marwali, Yoel Purnama, Santi Rahayu Dewayanti, A Ardiyan, Dafsah Arifa Juzar, Debby Siagian, Yih-Sharng Chen, Amer Aldhalia, Mark Ogino, Prashant Nasa, Christina Matthew, Nimisha Abdul Majeed, Wael Hafez, Indrek Ratsep, Andra-Maris Post, Piret Sillaots, Anneli Krund, Merili-Helen Lehiste, Tanel Lepik, Frank Manetta, Effe Mihelis, Iam Claire Sarmiento, Mangala Narasimhan, Michael Varrone, Mamoru Komats, Julia Garcia-Diaz, Catherine Harmon, S Veena Satyapriya, Amar Bhatt, Nahush A Mokadam, Alberto Uribe, Alicia Gonzalez, Haixia Shi, Johnny McKeown, Joshua Pasek, Juan Fiorda, Marco Echeverria, Rita Moreno, Bishoy Zakhary, Marco Cavana, Alberto Cucino, Giuseppe Foti, Marco Giani, Benedetta Fumagalli, Davide Chiumello, Valentina Castagna, Andrea Dell'Amore, Paolo Navalesi, Hoi-Ping Shum, Alain Vuysteke, Asad Usman, Andrew Acker, Benjamin Smood, Blake Mergler, Federico Sertic, Madhu Subramanian, Alexandra Sperry, Nicolas Rizer, Erlina Burhan, Menaldi Rasmin, Ernita Akmal, Faya Sitompul, Navy Lolong, Bhat Naivedh, Simon Erickson, Peter Barrett, David Dean, Julia Daugherty, Antonio Loforte, Irfan Khan, Mohammed Abraar Quraishi, Olivia DeSantis, Ahmad Nasrallah, Dominic So, Darshana Kandamby, Jose M Mandei, Hans Natanael, Ekya YudhaLantang, Anastasia Lantang, Surya Oto Wijaya, Anna Jung, George Ng, Wing Yiu Ng, Pauline Yeung Ng, Shu Fang, Alexis Tabah, Megan Ratcliffe, Maree Duroux, Ahmed Alajeeli, Ali Tarhabat, Shingo Adachi, Shota Nakao, Pablo Blanco, Ana Prieto, JesŁs Sanchez, Meghan Nicholson, Warwick Butt, Alyssa Serratore, Carmel Delzoppo, Pierre Janin, Elizabeth Yarad, Richard Totaro, Jennifer Coles, Bambang Pujo, Robert Balk, Andy Vissing, Esha Kapania, James Hays, Samuel Fox, Garrett Yantosh, Pavel Mishin, Safia Adem, Saptadi Yuliarto, Kohar Hari Santoso, Susanthy Djajalaksana, Arie Zainul Fatoni, Masahiro Fukuda, Keibun Liu, Paolo Pelosi, Denise Battaglini, Chiara Robba, Juan Fernando Masa Jimenez, Diego Bastos, Sérgio Gaião, Desy Rusmawatiningtyas, Young-Jae Cho, Su Hwan Lee, Tatsuya Kawasaki, Laveena Munshi, Pranya Sakiyalak, Prompak Nitayavardhana, Mohamed Bashir Elagili, Talat Ahmed Abu Salem, Tamara Seitz, Rakesh Arora, David Kent, Daniel Marino, Swapnil Parwar, Andrew Cheng, Jennene Miller, Shigeki Fujitani, Naoki Shimizu, Jai Madhok, Clark Owyang, Hergen Buscher, Claire Reynolds, Abusalama Abdurraouf, Ali Abdulnasir Kredan, Abdurrahman Haddud, Saad Moharam, Olavi Maasikas, Aleksan Beljantsev, Vladislav Mihnovits, Takako Akimoto, Mariko Aizawa, Kanako Horibe, Ryota Onodera, Carol Hodgson, Aidan Burrell, Meredith Young, Timothy George, Kiran Shekar, Niki McGuinness, Lacey Irvine, Brigid Flynn, Abdulrahman Almjersah, Tomoyuki Endo, Kazuhiro Sugiyama, Keiki Shimizu, Eddy Fan, Kathleen Exconde, Shingo Ichiba, Muhannud Binnawara, Hussein Embarek, Leslie Lussier, Gösta Lotz, Maximilian Malfertheiner, Lars Maier, Esther Dreier, Neurinda Permata Kusumastuti, Colin McCloskey, Al-Awwab Dabaliz, Tarek B Elshazly, Josiah Smith, Konstanty S Szuldrzynski, Piotr Bielaski, Yusuff Hakeem, Keith Wille, Srinivas Murthy, Ken Kuljit S Parhar, Kirsten M Fiest, Cassidy Codan, Anmol Shahid, Mohamed Fayed, Timothy Evans, Rebekah Garcia, Ashley Gutierrez, Hiroaki Shimizu, Tae Song, Rebecca Rose, Suzanne Bennett, Denise Richardson, Giles Peek, Lovkesh Arora, Kristina Rappapport, Kristina Rudolph, Zita Sibenaller, Lori Stout, Alicia Walter, Daniel Herr, Nazli Vedadi, Robert Bartlett, Antonio Pesenti, Shaun Thompson, Julie Hoffman, Xiaonan Ying, Bailey Williams, Emely Sanchez, Chika Akwani, Ryan Kennedy, Muhammed Elhadi, Matthew Griffee, Mary Mone, Anna Ciullo, Yuri Kida, Ricard Ferrer Roca, JordI Riera, Sofia Contreras, Cynthia Alegre, Christy Kay, Irene Fischer, Elizabeth Renner, Hayato Taniguci, James Lee, Daniel Plotkin, Barbara Wanjiru Citarella, Laura Merson, Emma Hartley, Bastian Lubis, Takanari Ikeyama, Alshaymaa Mortada, Ameen Alhamad, Ahmed Mechi, Islam Mohsen Ali Mohamed Hassan Nadar, Mohammed Saleh Alyasiri, Muhammed Zainab Alghali Elsaid, Balu Bhaskar, Jae-Seung Jung, Shay McGuinness, Glenn Eastwood, Sandra Rossi Marta, Fabio Guarracino, Stacy Gerle, Emily Coxon, Bruno Claro, Wafa Aldressi, Mahmoud Eleisawy, Hasnaa Osama, Daniel Loverde, Namrata Patil, Vieri Parrini, Angela McBride, Kathryn Negaard, Angela Ratsch, Ahmad Abdelaziz, Juan David Uribe, Adriano Peris, Mark Sanders, Dominic Emerson, Muhammad Kamal, Hamza Faida, Pedro Povoa, Roland Francis, Ali Cherif, Sunimol Joseph, Matteo Di Nardo, Micheal Heard, Kimberly Kyle, Ray A Blackwell, Amel Ouyahia, Michael Piagnerelli, Patrick Biston, Hye Won Jeong, Reanna Smith, Yogi Prawira, Giorgia Montrucchio, Arturo Huerta Garcia, Nahikari Salterain, Bart Meyns, Muhammed Elnasser, Marsha Moreno, Rajat Walia, Amit Mehta, Annette Schweda, Melissa Williams, Emad Amkhatirah, Kyung Hoon Kim, Alexandra Assad, Estefania Giraldo, Wojtek Karolak, Martin Balik, Elizabeth Pocock, Akram Mohamed, Evan Gajkowski, Mohamed Bedair, Kanamoto Masafumi, Nicholas Barrett, Yoshihiro Takeyama, Sunghoon Park, Faizan Amin, Fina Meilyana Andriyani, Serhii Sudakevych, Janos Schnur, Angela Ratsch, Magdalena Vera, Rodrigo Cornejo, Patricia Schwarz, Ana Carolina Mardini, Thais de Paula, Ary Serpa Neto, Andrea Villoldo, Alexandre Siciliano Colafranceschi, Alejandro Ubeda Iglesias, Juan Granjean, Lívia Maria Garcia Melro, Giovana Fioravante Romualdo, Diego Gaia, Helmgton Souza, Filomena Galas, Rafael Mez Mendiluce, Alejandra Sosa, Ignacio Martinez, Hiroshi Kurosawa, Mohammad Badr Almoshantaf, Juan Salgado, Beate Hugi-Mayr, Eric Charbonneau, Vitor Salvatore Barzilai, Veronica Monteiro, Rodrigo Ribeiro de Souza, Michael Harper, Hiroyuki Suzuki, Celina Adams, Jorge Brieva, Almu'atasim Khamees, Fadi Graige, Moh Supriatna, George Nyale, Faisal Saleem Eltatar, Jihan Fatani, Husam Baeissa, Ayman AL Masri, Ahmed Rabie, Mok Yee Hui, Masahiro Yamane, Hanna Jung, Ayorinde Mojisola Margaret, Newell Nacpil, Katja Ruck, Rhonda Bakken, Claire Jara, Tim Felton, Lorenzo Berra, Bobby Shah, Arpan Chakraborty, Monika Cardona, Gerry Capatos, Bindu Akkanti, Abiodun Orija, Harsh Jain, Asami Ito, Brahim Housni, Sennen Low, Koji Iihara, Joselito Chavez, Kollengode Ramanathan, Gustavo Zabert, Krubin Naidoo, Ian Seppelt, Marlice VanDyk, Sarah MacDonald, Shingo Ichiba, Randy McGregor, Teka Siebenaler, Hannah Flynn, Kristi Lofton, Toshiyuki Aokage, Bakar Kvirkvelia, Kazuaki Shigemitsu, Andrea Moscatelli, Giuseppe Fiorentino, Matthias Baumgaertel, Serge Eddy Mba, Jana Assy, Amelya Hutahaean, Holly Roush, Kay A Sichting, Francesco Alessandri, Debra Burns, Taha Husayn Alkhubouli, Ahmad Nasrallah, Ahmed Rabie, Gavin Salt, Carl P Garabedian, Jonathan Millar, Malcolm Sim, Adrian Mattke, Danny McAuley, Jawad Tadili, Tim Frenzel, Amro Abuleil, Yaron Bar-Lavie, Aaron Blandino Ortiz, Jackie Stone, Alexis Tabah, Antony Attokaran, Michael Farquharson, Brij Patel, Derek Gunning, Kenneth Baillie, Pia Watson, Kenji Tamai, Gede Ketut Sajinadiyasa, Dyah Kanyawati, Marcello Salgado, Assad Sassine, Bhirowo Yudo, Scott McCaul, Bongjin Lee, Sang Min Lee, Arnon Afek, Shimaa E Fattouh, Yoshiaki Iwashita, Hammad Fadlalmola, Bambang Pujo Semedi, Neurinda Permata Kusumastuti, Noureldin Mohamed Mansour, Jack Metiva, Nicole Van Belle, Ignacio Martin-Loeches, Mohammed Al-Sadawi, Cenk Kirakli, Al-Touny Shimaa, Lenny Ivatt, Chia Yew Woon, Hyun Mi Kang, Timothy Smith, Erskine James, Nawar Al-Rawas, Yudai Iwasaki, Hamza Ashour, Kenny Chan King-Chung, Vadim Gudzenko, Beate Hugi-Mayr, Fabio Taccone, Fajar Perdhana, Yoan Lamarche, Joao Miguel Ribeiro, Nikola Bradic, Klaartje Van den Bossche, Oude Lansink, Gurmeet Singh, Gerdy Debeuckelaere, Henry T Stelfox, Cassia Yi, Jennifer Elia, Thomas Tribble, Shyam Shankar, Raj Padmanabhan, Bill Hallinan, Luca Paoletti, Yolanda Leyva, Tatuma Fykuda, Jenelle Badulak, Jillian Koch, Lisa Janowaik, Amy Hackman, Deb Hernandez, Jennifer Osofsky, Katia Donadello, Aizah Lawang, Josh Fine, Benjamin Davidson, Andres Oswaldo Razo Vazquez, Ibrahim Abdehaleem

**Affiliations:** 1 Critical Care Research Group, The Prince Charles Hospital, Brisbane, Australia; 2 Faculty of Medicine, The University of Queensland, Brisbane, Australia; 3 PATH, Seattle, Washington, USA; 4 Leap Ahead GmbH, Emsdetten, Germany; 5 Australian Centre for Health Services Innovation, Queensland University of Technology, Brisbane, Australia; 6 Queensland University of Technology, Brisbane, Australia; 7 St Andrew’s War Memorial Hospital, UnitingCare Hospitals, Brisbane, Australia; 8 Wesley Medical Research, Brisbane, Australia; 9 School of Medicine, Griffith University, Brisbane, Australia

## Abstract

**Background:**

The global scarcity of medical oxygen has proven to be catastrophic during the surges in COVID-19 cases over the past two years, with the heaviest burden felt in low- and middle-income countries. Despite its criticality, data and analyses of oxygen consumption, even for typical clinical cases, are missing. Consequently, planning oxygen needs, particularly with variable surges in COVID-19 cases, has presented a substantial challenge to policymakers and hospital decision-makers.

**Methods:**

We performed a sub-analysis of the COVID-19 Critical Care Consortium database assessing the oxygen consumption requirements of COVID-19 patients admitted to intensive care units between February 2020 and October 2021. We calculated descriptive statistics for oxygen flow-rates, stratified by oxygen supplementation method, and developed a multi-state model for estimating the frequency, therapy duration, probability of transition, and number of oxygen therapy modes per patient.

**Results:**

Overall, 12 429 patients from 35 countries received oxygen support on at least one day of their hospitalisation. Of the patients with measurable flow rates, 6142 received invasive mechanical ventilation, 838 received high-flow nasal oxygen, and 257 received both modalities. The median flow rate for mechanical ventilation was 3.2 L per minute (interquartile range (IQR) = 2.0–4.9), with a median duration of 12 days (IQR = 6–24), while the median flow rate for high-flow nasal cannula was 40 L per minute (IQR = 15–55), with a median duration of three days (IQR = 2-6).

**Conclusions:**

Oxygen consumption among critical COVID-19 patients varies by mode of delivery (invasive ventilation vs high-flow nasal cannula), across patients, and over treatment duration. Therefore, it is essential that health facilities routinely monitor oxygen utilization to better inform oxygen delivery system design and regular supply planning.

**Registration:**

ClinicalTrials.gov: CTG2021-01 ACTRN12620000421932.

Oxygen therapy has been a first-line treatment for severe and critical coronavirus disease 2019 (COVID-19) infection since the start of the pandemic [1]. Optimal delivery of supplemental oxygen follows a step-wise approach with continuous assessment of patient response to therapy and generally operates under the assumption that the supply of medical oxygen is not a constraint in escalating treatment. British Thoracic Society guidelines offer a standard approach, ensuring the judicious application of resource-intensive oxygen therapies such as high-flow nasal oxygen or mechanical ventilation [2]. For the most part, these guidelines do not consider oxygen supply as a limiting resource and instead consider limits on nursing, medical, and technical support.

As the COVID-19 pandemic surged, weaknesses in global oxygen supply chains became apparent, leading to scarcity of medical oxygen. While it quickly became clear that low- and middle-income countries (LMICs) had a profound lack of oxygen [3–5], high-income countries with established methods for supplying oxygen also struggled to meet patient demand during COVID-19 surges [6,7]. In this context, the lack of oxygen became a limitation to the provision of care. This was further exacerbated by the fact that oxygen value chains are complex and rely on heavy infrastructure that cannot be easily planned for, nor scaled in a short timeframe. In most high-income countries, health facilities can rely on large industrial gas manufacturers that produce liquid oxygen, and transport and store it in cryogenic storage tanks at facilities. On demand, liquid oxygen is vaporised and travels through a piping system to a wall outlet, where health practitioners can plug in delivery devices accommodating different treatment methods (masks, nasal prongs, ventilators, high-flow nasal cannulas, etc). While these systems are well-established in high-income countries, the tremendous oxygen needs that some countries faced outstripped supply. Many LMICs only partially (or not at all) rely on a stable liquid oxygen value chain. Alternative modes of production are for example pressure-swing adsorption plants that produce gaseous oxygen. Some facilities have a piping system in place to deliver oxygen to a patient’s bedside, but in most cases, oxygen is filled into cylinders and transported to a health facility. There they are, in some cases, connected to a small piping system via a cylinder manifold; if such a system is not available, they are carried to a patient’s bed to deliver oxygen. An alternative to this are oxygen concentrators that are sometimes used to produce oxygen at a patient’s bedside and which directly serve the patient’s oxygen needs [8].

Consequently, new methods for estimating oxygen supply were required to plan and scale up oxygen systems as a response effort. In the absence of substantiated evidence on oxygen demand for COVID-19 patients, planners had to rely on rough estimates. For example, the British Health Technical Memorandum indicate to plan for a bed with ten litres per minute (LPM). For a few other departments, they suggest higher design flow rates at 100 LPM. For example, rooms with continued-positive airway pressure ventilation should be planned for with 75 LPM, and anaesthetic and operating rooms at 100 LPM. Flow rates are reduced for additional beds added to the system to ensure that it is not completely oversized [9]. At the beginning of the pandemic, the World Health Organization (WHO) also put forward a planning tool to help low- and middle-income countries (LMICs) estimate oxygen system requirements [10]. These estimates state that a severely ill COVID-19 patient requires 10 LPM for seven days, while a critically ill one requires 30 LPM for 14 days [1].

These estimates led to significant debates among planners. Adding the high flow rate estimates for individuals to size the oxygen requirements of a ward raised concerns about over-sizing oxygen systems. Anecdotes suggested that far lower averages would be used, particularly in low-resource settings, where over-sizing systems would ultimately result in limiting access if scarce financial resources are only sufficient for a few facilities. Conversely, others argued that patients require different flow rates and that oxygen systems require buffers to deal with these fluctuations. The discussion showed that lacking data on a new disease such as COVID-19 creates substantial challenges to planning and resourcing in particular if these activities are inflicted with time pressure during a pandemic response.

As the pandemic progressed, several avenues were explored to refine estimates of COVID-19 patients’ oxygen consumption. These included studies focused on individualising oxygen requirements based on clinical and laboratory variables, studies retrospectively analysing site-specific oxygen utilisation in high-income countries, and an upcoming clinical trial which will quantify oxygen utilisation at the facility level in LMICs [11–13]. We contribute to this stream of research by analysing the oxygen consumption of COVID-19 patients admitted to intensive care units (ICUs) using data from 36 countries of varying economic status. Our objective was to analyse oxygen flow rates and therapy duration for patients with COVID-19, informed by the diverse and highly granular data within the COVID-19 Critical Care Consortium database to better understand the flow rate patterns and variability, and to compare observed consumption to planning data.

## METHODS

### Study design

In this retrospective observational study of oxygen consumption, we used a data set provided by the COVID-19 Critical Care Consortium, which is an international multi-centre observational study with 403 collaborating centres across 53 countries [14]. Participating sites gather daily data from hospital admission to discharge using a combination of data collection tools from the International Severe Acute Respiratory and emerging Infection Consortium/Short PeRiod IncideNce sTudy of Severe Acute Respiratory Infection (ISARIC/SPRINT-SARI) (beginning at hospital admission) and the Consortium (beginning at ICU admission) [15]. Participating hospitals obtained approval from local ethics committees. A waiver of informed consent was granted for collection of de-identified patient data which were stored using the REDCap electronic capture systems hosted at the University of Oxford, United Kingdom, and at Monash University, Melbourne, Australia. The full study protocol has been previously published elsewhere [14].

### Study population

All patients admitted to an ICU with COVID-19 between 1 November 2019 and 31 October 2021 that received oxygen via mechanical ventilation or high-flow nasal cannula (HFNC) on at least one day of their hospital admission were included. For patients with multiple ICU admissions, only the data for the first admission was recorded. Both patients with laboratory confirmed diagnosis or high clinical suspicion were included, such that patients enrolled in countries without laboratory testing capacity were not unduly excluded. Patient data were collected intermittently dependent on the daily availability of data. At a minimum, the case-report form required data entry on key dates of the patient’s ICU stay when major treatment variables changed (i.e. commencement and cessation of mechanical ventilation, hospital discharge, etc.). Additional days of data were entered for some patients depending on site resource availability.

### Derivation of oxygen therapy duration and transitions

We defined a multi-state model with five states estimate oxygen therapy duration and transitions in the context of intermittent data observations. The three transition states were being hospitalised without oxygen therapy, receiving high-flow nasal oxygen therapy, and receiving mechanical ventilation. The two absorbing states were hospital discharge or death. The multi-state model was developed according to the previously published method for panel data [16]. We calculated sojourn times, total length of stay per state, number of transitions, and transition probabilities and derived 95% confidence intervals. This method allowed for interpolation of treatment duration despite the intermittent and inconsistent observation times between patients.

### Derivation of oxygen flow rates

The study case-report form included patients receiving either HFNC or mechanical ventilation. For the former group, the supplemental oxygen flow was reported in litres per minute (LPM). For the latter, the equivalent oxygen flow-rate requirement in LPM was approximated from the patient’s fraction of inspired oxygen (FiO_2_), i.e. the fraction of oxygen in the gas mixture supplied to a patient corrected for room oxygen, the respiratory rate, and the tidal volume according, to the below formula adapted from previous studies and from manufacturer advice [17,18].

Invasive ventilation LPM = respiratory rate (breath/min) × tidal volume (ml/breath) x 1/1000 (l/ml) x (FiO_2_ (%) − 21%)

Each of the variables used to approximate the flow-rate were recorded simultaneously once per day by data collectors. The case-report form specifies that these data are to be recorded at the time of peak FiO_2_ on that day, i.e. at the time of peak oxygen requirement.

We performed an additional analysis for patients that received both mechanical ventilation and HFNC during their admission by assessing average flow rate of HFNC at critical transition points (i.e. HFNC flow rates the day before and after mechanical ventilation was commenced). Derivation of oxygen flow rates from other forms of non-invasive ventilation was not feasible due to data availability and practical constraints on deriving flow rates from FiO_2_ with other modalities [19].

### Statistical analysis

We generated summary statistics for patient demographics, clinical characteristics, and outcomes. We reported continuous variables as medians with interquartile ranges (IQRs) to allow for consistent representation of data regardless of normality, and categorical variables as numbers with percentages. We used Kolmogorov-Smirnov tests to check for normality of data distribution (Table S2 in the **Online Supplementary Document**). We generated descriptive statistics for the flow rate of oxygen with stratification by treatment method. For population summary statistics, we calculated the average flow-rate per patient for the duration of the hospital stay. We applied multi-state modelling to derive the total duration of oxygen therapy per patient, sojourn times when different oxygen delivery modes are used, and for assessing the likelihood of transitioning between oxygen therapy modes. This approach was used because, for most patients, data entry was only completed on key days of the hospital stay dependent on the version of case-report form completed.

## RESULTS

### Patient demographics

As of 20 October 2022, there were 16 232 patients in the database from 262 recruiting sites across 36 countries which fell within the inclusion date range (1 November 2019 to 31 October 2021). An additional 681 patient entries were present in the database, but were excluded due to having incomplete data (i.e. no other data except a patient identifier) or being otherwise ineligible for inclusion (i.e. inclusion criteria and essential dates outside of feasible range).

**Figure 1:**
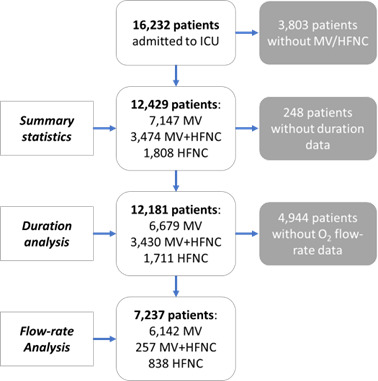
Patient enrolment flowchart including exclusions.

Of these 16 232 patients, 12 429 from 35 countries met the inclusion criteria of receiving mechanical ventilation or HFNC oxygen. Among them, 7147 received solely mechanical ventilation, 3474 received mechanical ventilation and HFNC throughout their stay, and 1808 received solely HFNC (**Figure 1**, **Table 1**). Overall, 10 793 (86.8%) were from 23 high income countries, 1417 (11.4%) were from 10 upper-middle income countries, and 219 (1.8%) were from 2 low-income countries, according to World Bank definitions [20].

**Table 1:** Descriptive statistics of patient population

	MD (IQR)	Mean	Missing, n (%)
**Age in years**	61 (51–69)	59	5 (0.1)
**BMI in kg/m^2^**	28.6 (25.7–32.7)	29.9	1692 (7.3)
**SOFA score**	5 (3–8)	5.9	9967 (66.9)
**SpO_2_ in %**	93 (88–96)	90.0	2748 (12.4)
**Respiratory rate in breaths/min**	25 (20–30)	26.2	4030 (19.3)
**Length of hospital stay in days**	19.5 (10–35)	27.1	7127 (40.2)

BMI – body mass index, IQR – interquartile range, MD – median, SOFA – sequential organ failure assessment, SpO_2_ – oxygen saturation, x̄ – mean

**Figure 2:**
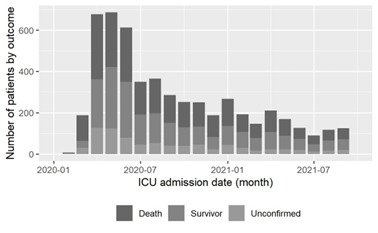
Outcome-stratified patient enrolment numbers over the duration of the study period.

At the time of analysis, 2522 patients were reported as having died during their COVID-19 hospitalisation; 5867 were discharged from hospital (43.0% mortality rate); 280 were still in hospital; 24 were discharged to palliative facilities; and 3161 had an unconfirmed outcome status. Patient mortality was the highest in the early months of the pandemic (67.0% in March and April 2020) with an overall trend of decreasing patient mortality over time (**Figure 2**).

### Oxygen flow rates

**Figure 3:**
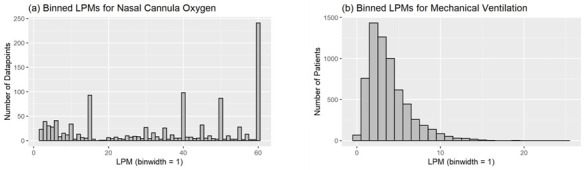
Distribution of oxygen flow rates by mode.

Of the 12 429 patients who received oxygen therapy on at least one day of their hospital admission, 7251 had at least one day where the flow rate of oxygen could be determined (**Table 2**). For each patient, the average flow rate for mechanical ventilation or HFNC is calculated for the duration of their admission. The data indicates that 50.0% of patients treated with oxygen supplied by HFNC require less than 40 LPM of oxygen. The highest flow rate we observed was 60 LPM. The distribution of flow rates across the spectrum was non-uniform, with peaks at major flow rate settings (15, 40, 45, and 60 LPM in particular). The data for mechanical ventilation showed that 50.0% of the patients were receiving around 3.2 LPM of oxygen or less. The other half of the patients received higher flows of oxygen up to 24.8 LPM (**Figure 3**, Panels A–B).

**Table 2:** Descriptive statistics of average oxygen flow rates in LPM for different ventilation methods

	n	MD (IQR)	x̄ (SD)	Maximum
**HFNC**	1095	40 (15.0–55.0)	34.8 (20.7)	60
**Mechanical ventilation**	6399	3.2 (2.0–4.9)	3.8 (2.5)	24.8

HFNC – high-flow nasal cannula, MD – median, SD – standard deviation, x̄ – mean

**Figure 4:**
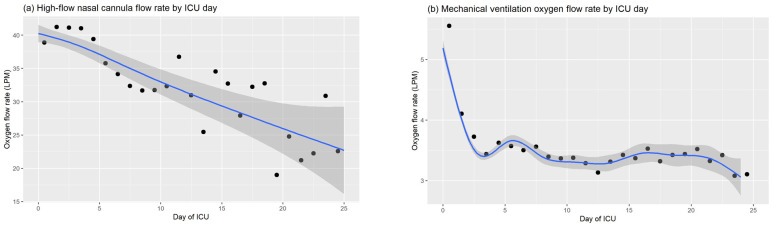
Mean flow rates with 95% confidence intervals (shaded) graphed by day of intensive care unit stay.

Aggregated by day of ICU stay, the average flow-rates and confidence intervals are shown in Figures 4a and 4b. It is interesting to observe that over the course of ventilation the oxygen flow-rates drop. This is based in clinical reasoning, where patients in severe respiratory distress enter the ICU and receive a high flow of oxygen over the first hours and days of treatment to stabilize them, and are slowly weaned over the following days. It is also worth noting that according to our averaged data the rate at which oxygen is reduced is different across ventilation methods. While mechanically ventilated patients receive on average a higher dose only on the first day, the data suggests that HFNC ventilated patients require not only more oxygen but also they remain on a higher level of oxygen for a longer time period.

### Treatment duration and treatment pathways

**Figure 5:**
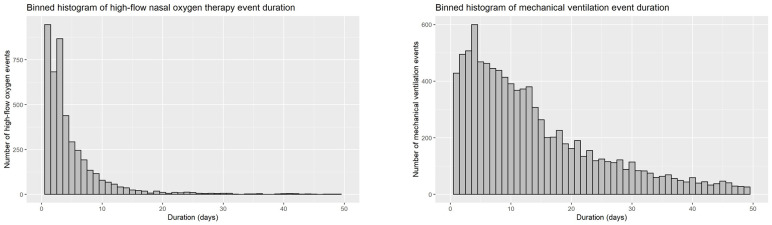
Distribution of oxygen treatment duration by delivery mode.

Sojourn times were considered to capture the durations of oxygen administration per treatment rather than per patient (i.e. a patient bridged to mechanical ventilation with HFNC that was weened again with HFNC would have had two separate HFNC treatment durations) (**Table 3**). The results showed that 50.0% of the patients entering HFNC treatment remained on the cannula for three days or less. Patients on mechanical ventilation spent on average far more time on oxygen therapy; 50% of them spent 12 days or less on oxygen (**Figure 5**).

**Table 3:** Descriptive statistics of treatment duration in days for different ventilation methods

	n	MD (IQR)	x̄ (SD)
**HFNC**	4552	3 (2–6)	6.98 (13.3)
**Mechanical ventilation**	10 460	12 (6–24)	19.1 (20.3)

HFNC – high-flow nasal cannula, MD – median, SD – standard deviation, x̄ – mean

**Figure 6:**
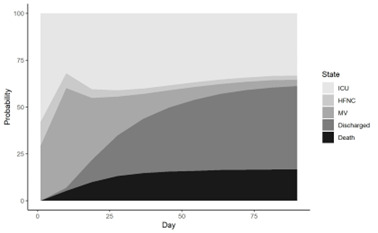
State probability table showing observed percentages of states over time. The vertical slice provides the probability of being in one of the five states.

Regarding the state probabilities over the ICU admission period (**Figure 6**), the probability of patients being in the state of discharge or death naturally increased as treatment progresses. The probability of being in the state of non-ventilated ICU remained somewhat stable. Notably, the probability of patients being on HFNC stays somewhat stable indicating that a certain proportion of patients are typically on HFNC or weened-off with HFNC, while the probability of being on mechanical ventilation declines significantly (**Figure 6**).

There were a total of 1602 patients who received HFNC immediately before transitioning to mechanical ventilation. The median duration of HFNC prior to mechanical ventilation commencement was two (IQR = 1–5). There were 1180 cases where HFNC commencement immediately followed a period of mechanical ventilation, with the median duration of HFNC in these instances being three days (IQR = 1–7).

**Figure 7:**
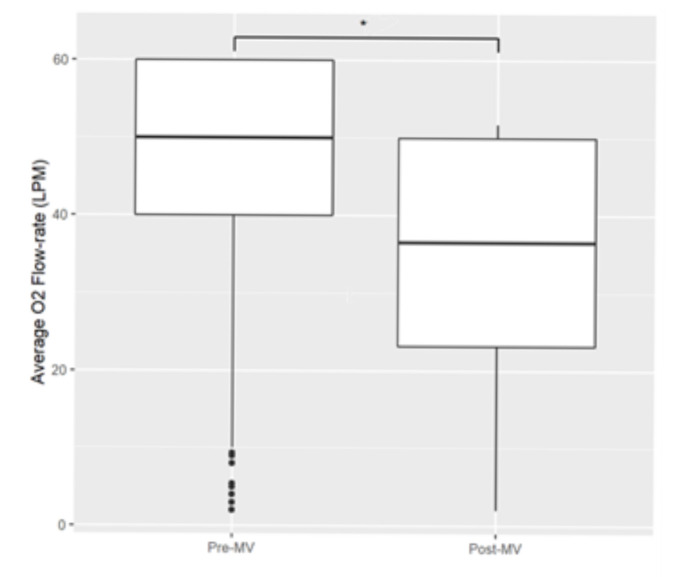
Boxplot of HFNC flow rates pre- and post-mechanical ventilation. The boxplot summarises the average flow rates per patient during the respective periods in litres per minute. *P = 0.01 using Welch’s unequal variances *t*-test.

To further explore the role of HFNC ventilation, we examined the oxygen consumption of patients on HFNC pre- and post-mechanical ventilation (**Figure 7**). A total of 134 patients had data on HFNC flow rate before or after mechanical ventilation. The median flow rate pre-mechanical ventilation was 50 LPM (IQR = 40–60), while the median flow rate post-mechanical ventilation was significantly lower at 37 LPM (IQR = 22–50) (Welch’s unequal variances *t*-test *P* = 0.01).

## DISCUSSION

In this large, international, multi-centre observational study, we characterised oxygen flow rate requirements with a median of 3.2 LPM for invasive mechanical ventilation (IQR = 2.0–4.9) and a median of 40 LPM for high-flow nasal oxygen (IQR = 15–55). We determined a median period of oxygen therapy of 12 days for invasive mechanical ventilation (IQR = 6–24) and of 3 days for high-flow nasal oxygen (IQR = 2–6).

Our results show that flow rates vary substantially by patient over the course of treatment as well as by ventilation method, indicating how difficult it is to estimate oxygen flow rates, even if the clinical profile of patients is specific (in this case, COVID-19 patients). Compared to a single-centre study by Hvarfner et al. [11] similarly exploring oxygen flow rates for COVID-19 patients admitted to the ICU, we found higher flow rates and longer treatment durations for COVID-19 patients receiving supplemental oxygen via mechanical ventilation. The aforementioned researchers [11] studied oxygen consumption in 126 patients at a Swedish district hospital and found a median of 2.0 LPM (IQR = 1.3–3.5). They also reported lower treatment durations of 2.3 days (IQR = 0.68–4.20). The oxygen treatment duration estimates for mechanical ventilation derived from our data seem more in line with findings from King et al. [21] who determined that, in 1023 patients, those that survived and died were intubated with an average duration of 14.6 days (IQR = 1–59 days) and 9.3 days, respectively. In a study of 72 COVID-19 patients, Ferguson et al. [22] found an average ICU stay to be 17 days (IQR = 13–29); meanwhile, in a study of 1000 COVID-19 patients, Argenziano et al. [23] determined a median duration of 9.0 days (IQR = 6.5–12.0) for discharged patients who received mechanical ventilation. To the best of our knowledge, our study is the first to characterise flow rates of COVID-19 patients receiving HFNC support.

Comparing the consumption-oriented flow rates in our study with planning figures underlines the higher needs in high-dependency units indicated by the planning figures. According to our analysis, the World Health Organization’s planning figures of 30 LPM for critically ill patients over 14 days of treatment and 10 LPM for severely ill patients over 7 days would cover almost 100% or 97% of the mechanical ventilation flow rate requirements, respectively, and 40% or 21% of the HFNC requirements (**Figure 3**, Panels A–B). Treatment duration estimates from our study seem to suggest that some patients stayed in the ICU considerably longer. The 7 and 14 days proposed by the WHO planning figures cover 33% and 58% of the treatment duration for mechanical ventilation and 80% and 92% for HFNC treatment. Comparing the flow rates to planning figures from the British standards, however, is not as straightforward. The standards put forth a flow rate of 10 LPM for a high-dependency care bed for the first bed, and further state that three-quarters of the other beds in a planning area must be able to deliver 6 LPM [9]. While 10 LPM would cover 94% of the patients in our study, it would drop to 83% for 6 LPM, ignoring any statistical aggregation effects in a ward (**Figure 3**, Panel B). The standards also highlight that, if oxygen is used to power ventilators or they are operating in a continuous positive airway pressure (CPAP) method, a far higher flow rate is required. The standard suggests that 75% of the beds should be able to deliver 75 LPM of oxygen. This planning figure would be appropriate for HFNC according to the numbers in our study, which we determined to be up to 60 LPM (**Figure 3**, Panel A).

Our findings indicate that mechanically ventilated COVID-19 patients required rather high flow rates and long treatment times. They also suggest that the internationally recognised planning figures provided by the two organisations would cover a large percentage of the oxygen consumption we found for mechanical ventilation and HFNC. Fluctuations in oxygen use necessitate a thoughtful matching of need with oxygen supply. This highlights that oxygen flow rates are not stable, and averages can be misleading for planning purposes. Our comparison of planning figures from internationally recognised organisations resulted in very high coverage of the continuum of flow rates, indicating that the flow rates they provided are not meant to be averages, but rather (close to) peak flows. Vice versa, it also means that planning the primary oxygen system against these numbers results in a large system that can cover almost all demand cases. While this is clinically desirable, in particular for low-resource settings, this may limit system-wide oxygen access, as a few large centralised systems at higher-level facilities may not reach the broader population distributed across an entire country. The results call to build in back-up systems to cover peaks and otherwise use a smaller scale, less expensive oxygen source. The large deviation of the empirical median from the planning figures indicates that arguments for using the median (or the average) to determine the system size are problematic, as they may fail to cover higher flow rates even if statistical aggregation effects are taken into account.

This study provides a first indication of how to calculate the frequency of near-peak oxygen use, as well as to predict how often one might surpass this peak under atypical circumstances like a pandemic or based simply on the delivery interface utilised (e.g. ventilator vs HFNC). It reinforces the individualisation in oxygen therapy and the advantages that come with flexibly increasing capacity as needed. Our findings also confirm the benefit of surge or back-up capacity to accommodate unanticipated increases in consumption as was the case during the pandemic. Decision-makers should be aware of this variability and consider concepts to plan accordingly to ensure sufficient oxygen provisions are available.

However, the oxygen requirements from our study (that is, a measure of consumption) are not entirely the same as planning figures that are supposed to help determine capacity or supply. Planning figures mandate to account for losses – for example, leaks in the piping system or losses when devices are switched. Also, a certain level of buffer should be available to ensure reliable supply; as such, it is acceptable if planning figures are higher than consumption-based estimates. Our direct comparison of patient flow-rate requirements to planning figures also does not account for statistical aggregation effects. That is, it is very unlikely that all patients in a ward require the maximum flow at the same time and as such a mere summation of peak flow will likely end up in an over-capacitated system.

Importantly, COVID-19 posed a new challenge to clinicians; over the course of the pandemic, treatment recommendations changed as clinicians collected evidence. For example, most patients within our cohort who received HFNC progressed to mechanical ventilation, implying that it may have been used as a bridging therapy or that patients were unlikely to receive extended HFNC in favour of mechanical ventilation. Moreover, the use of HFNC was discouraged due to the risk of aerosolisation of severe acute respiratory syndrome coronavirus 2 (SARS-CoV-2) and an increased risk of virus transmission. This theory was rooted largely in speculation, and evidence emerged which argued that HFNC has limited potential for increasing transmission rate [24]. In fact, the WHO released a preliminary recommendation suggesting to use HFNC, CPAP, and other non-invasive ventilation methods if the patient is not in immediate need of intubation [25]. This indicates that the HFNC treatment lengths we found may not be considered representative for a stand-alone treatment of COVID-19 patients with HFNCs.

### Limitations

The principal limitation of this study is the approximation of oxygen flow rates using the total oxygen consumption formula. The benefit of this approach is the potential to quantify individualised oxygen requirements, but there are several factors which limit its accuracy. Chiefly, we did not capture additional oxygen utilisation by the ventilator device for baseline flow or line leakage, which may make the practical oxygen requirements greater.

The major benefits of this study stem from the granular insights into the individual oxygen requirements of patients admitted to ICU. Where other studies are subject to the variable definitions of COVID-19 severity, our study instead focussed on patients who required high-level supportive care and monitoring, as defined by ICU admission. The caveat to this, however, is that we did not capture the broad range of oxygen delivery modalities which are used less frequently in ICU, including venturi masks, low-flow nasal prongs, and other forms of ward oxygen therapy. Additionally, we did not capture the variable criteria for ICU admission, nor the way that resource constraints throughout the pandemic period may have skewed the level of treatment received in ICU.

An additional limitation is that this study is based on the highest level of oxygen reported on each day. The case report form specifies to include the maximum FiO_2_ recorded on that day, usually correlating with the highest level of oxygen consumption. As such, these estimates are likely to be on the higher end of what is expected for average oxygen consumption per patient. This may explain why our estimates are greater than those reported in other studies.

Other factors to consider relate to the study period and breadth; here we summarised data for the first 19 months of the pandemic, which may not be reflective of the milder respiratory disease noted in more recent strains. We also did not capture the decision-making behind treatment variables, including the possibility that oxygen supplementation was dictated by supply in some regions throughout the study period, and we also did not account for changes in how oxygen was prescribed over the course of the reporting horizon.

## CONCLUSIONS

Our analysis revealed a substantial variance in the oxygen requirements within a ventilation method and across methods. We also noticed a strong variation in treatment length and average oxygen flow rates required over time. This evidence highlights that oxygen flow rates are not stable and that averages can be misleading for planning purposes. Decision-makers should be aware of this variability and consider concepts to plan accordingly to ensure sufficient oxygen provisions are available.

Further work is required to support better planning. In particular, resource-constrained settings in LMICs need to balance between sufficient availability of oxygen and the cost to sustain high availability. For decision-makers and facility managers in these contexts, understanding the nuanced use of oxygen is essential to making informed supply choices. This research, combined with other, forthcoming insights from the Lancet Global Health Commission on Medical Oxygen Security [26], will ensure these choices are made with cost efficiency in mind. Oxygen delivery technologies can accommodate variability in oxygen use to varying degrees. Liquid oxygen is more capable of responding to fluctuations in demand as users can increase the frequency of refills when demand spikes, whereas pressure swing adsorption is a fixed capacity with a known production limit.

Regardless of the oxygen source, this research demonstrates the value of better data visibility in patient health records and the supply system. We relied on a network of health facilities to track oxygen consumption for COVID-19 patients. More work should be done to understand oxygen requirements for other treatment indications and within other clinical settings to understand overall consumption. In LMICs, this will require investments in electronic or paper-based medical records, as well as consensus around standard indicators for tracking hypoxemia and oxygen utilisation. Besides patient-level data, data visibility can be improved in the supply chain for medical oxygen via remote monitoring.

## Additional material


Online Supplementary Document
