# Supplementary material for: Variability of oxygen requirements in critically ill COVID-19 patients
Source: J Glob Health. 2024 Feb 23;14:05012. doi: 10.7189/jogh.14.05012 (PMC10884784; doi:10.7189/jogh.14.05012)
Supplement: Online Supplementary Document [file jogh-14-05012-s001.pdf]

Figure S1. Diagram of states and transitions in the multi-state model.

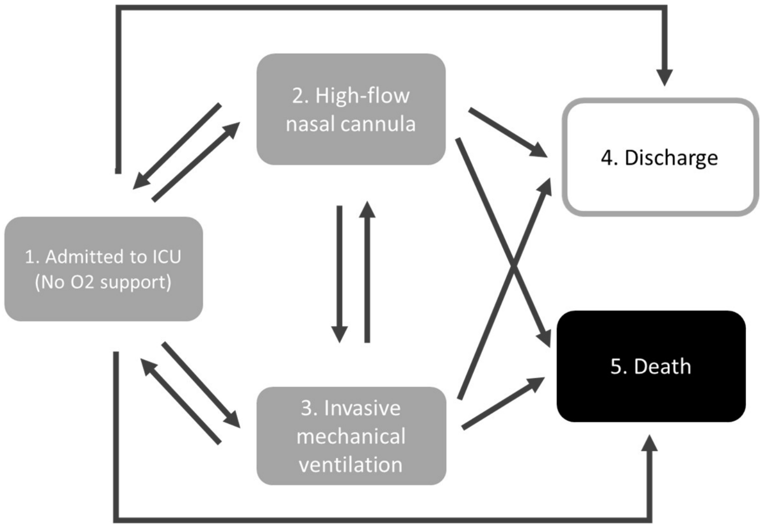

Table S1. List of contributors and collaborators in the COVID-19 Critical Care Consortium.

Contributors

Prefix/First Name/Last Name	Site Name
Tala Al-Dabbous<br>Huda Alfoudri<br>Mohammed Shamsah	Al Adan Hospital
Khadeejeh Alfroukh<br>Zinah Aqeel Abdulzahra Bairmani<br>Khalid Jehad Khalid<br>Salsabeel M.A. Abukhalaf	Al Ahli Hospital
Mohammed Maher Hadhoud	Al Menshawy General Hospital
Mohamed Fathi	Al-Hawari Center of Surgical Speciality
Hasan Alhouri	Al-Mouwasat University Hospital - Damascus
Dr Hamza Shahla	Al-Thawra Teaching Hospital in Albayda
Qamrah Alhadad<br>Matly Hanan	Aljufrah Clinical Isolation Center
Subbarao Elapavaluru<br>Ashley Berg<br>Christina Horn	Allegheny General Hospital
Ahmed Reda Mohamed Elsayed Abdelhalim<br>Amro Essam Amer<br>Cinderella Omar Rageh Elnaggar<br>Ahmed Ayman Hassan<br>Ali Abdelaziz<br>Mohamed Abdelhalim<br>Yehia Samir Shaaban Aly Orabi	Alexandria Main University Hospital
Zinah A. Alaraji<br>Mo'nes R. Muhaisen<br>Lana Almasri<br>Dana Mustafa<br>Shaher Hamdan<br>Yousef Al-Saba'a<br>Zaina Dalloul<br>Mohammed Alkahlout<br>Hamza Jaber<br>Osama Aldabbourosama<br>Alaa Abdalfattah Abdalhadi	Alshifa Hospital, Gaza
Aliae AR Mohamed Hussein<br>Zarief Kamel Emad	Assiut University Hospital

Sarah Khaled<br>Nouralsabah Mohamed<br>Ebtisam Hassanin<br>Abdelhafeez Hamdi<br>May Gamal<br>Ahmed Emad<br>Abdelrahman Ragab<br>Mohammed G Azizeldin	
Almthani Hamza<br>Alsarrah Ali Mohammed Omer<br>Asgad Osman Abdalla Fadl alla<br>Asia Atif Abdelrahman Abdallahrs<br>Aml Ahmed Eltayeb<br>Maali khalid mohamed abdalla<br>Alhasan<br>Esraa Hassan Abdelgaum<br>Aya Mustafa Ahmed<br>Lamees Adil Abdulbaqi<br>Omer Abdullah Mohammedelhassan<br>Musaab Mohammed Mohammed<br>Ahmed<br>Maha TagElser Mohammed Ali	Atbara Teaching Hospital
Yunis Mayasi	Avera McKennan Hospital & University Health Centre
Stephan Schroll	Barmherzige Bruder Regansburg
Dan Meyer<br>Jorge Velazco<br>Ludmyla Ploskanych<br>Wanda Fikes<br>Rohini Bagewadi<br>Marvin Dao<br>Haley White<br>Alondra Berrios Laviena<br>Ashley Ehlers<br>Maysoon Shalabi-McGuire<br>Trent Witt	Baylor Scott & White Health
Lorenzo Grazioli<br>Luca Lorini	Bergamo Hospital
E. Wilson Grandin<br>Jose Nunez<br>Tiago Reyes	Beth Israel Deaconess Medical Centre
Diarmuid O'Briain<br>Stephanie Hunter	Box Hill Hospital
Mahesh Ramanan<br>Julia Affleck	Caboolture Hospital
Hemanth Hurkadli Veerendra<br>Sumeet Rai<br>Josie Russell-Brown<br>Mary Nourse	Canberra Hospital
Mark Joseph<br>Brook Mitchell<br>Martha Tenzer	Carilion Clinic

Ryuzo Abe	Chiba University Graduate School of Medicine
Hwa Jin Cho<br>In Seok Jeong	Chonnam National University Hospital
Nadeem Rahman<br>Vivek Kakar<br>Ahmed Tamimi<br>Diala Zabalawi<br>Mohamed Elhennawi<br>Praveen Ghisulal<br>Sadaf Malik	Cleveland Clinic- Abu Dhabi
Nicolas Brozzi	Cleveland Clinic - Florida
Omar Mehkri<br>Sudhir Krishnan<br>Abhijit Duggal<br>Stuart Houltham	Cleveland Clinic - Ohio
Jerónimo Graf	Clinica Alemana De Santiago
Roderigo Diaz<br>Roderigo Orrego<br>Camila Delgado<br>Joyce González<br>Maria Soledad Sanchez<br>Michael Piagnerelli<br>Josefa Valenzuela Sarrazin	Clinica Las Condez
A/Prof. Gustavo Zabert<br>Lucio Espinosa<br>Paulo Delgado<br>Victoria Delgado	Clinica Pasteur National- University of Comahue
Diego Fernando Bautista Rincón<br>Angela Maria Marulanda Yanten<br>Melissa Bustamante Duque	Clinica Valle de Lilli
Daniel Brodie	Medical ICU, Columbia College of Physicians and Surgeons, New-York-Presbyterian Hospital, NY, NY, USA
Khaled Abouelmagd	Dr. Mohammad Alfagih Hospital
Alyaa Elhazmi<br>Abdullah Al-Hudaib	Dr Sulaiman Alhabib Medical Group – Research Center, Riyadh, Saudi Arabia
Jeff Javidfar<br><br>Maria Callahan<br>Andy Dong<br>Charles Crepy D'Orleans	Emory University Healthcare System
M. Azhari Taufik<br>Elizabeth Yasmin Wardoyo<br>Margaretha Gunawan<br>Nurindah S Trisnaningrum<br>Vera Irawany<br>Muhammad Rayhan	Fatmawati Hospital
Mauro Panigada<br>Antonio Pesenti<br>Alberto Zanella<br>Giacomo Grasselli<br>Sebastiano Colombo	Fondazione IRCCS Policlinico of Milan (Fondazione IRCCS Ca' Granda Ospedale Maggiore Policlinico)

Chiara Martinet<br>Gaetano Florio	
Massimo Antonelli<br>Simone Carelli<br>Domenico L. Grieco	Fondazione Policlinico Universitario Agostino Gemelli IRCCS
Motohiro Asaki	Fujieda Municipal General Hospital
Kota Hoshino	Fukuoka University
Leonardo Salazar<br>Mary Alejandra Mendoza Monsalve	Fundación Cardiovascular de Colombia
John Laffey<br>Bairbre McNicholas<br>David Cosgrave<br>Minha Atif<br>Fadi Qutishat<br>Caoimhe Laffey<br>Michaeal Van Der Walt	Galway University Hospitals
Joseph McCaffrey<br>Allison Bone<br>Jemma Trickey<br>Michelle Horton<br>Michelle Horton<br>Stephanie Pearce<br>Tania Salerno	Geelong Hospital
Akram Mohamed<br>Salem Alhaddad<br>Baliad Bakeer<br>Shames Haitam<br>Laila Shalabi<br>Mohammed Abodina Ahmed	Gheryan Central Hospital
Yusuff Hakeem	Glenfield Hospital
James Winearls<br>Mandy Tallott	Gold Coast University Hospital
David Thomson<br>Ivan Joubert<br>Christel Arnold-Day<br>Jenna Piercy<br>Richard van Zyl Smit<br>Malcom Miller<br>Lisa Seymour<br>Francois van Heyningen<br>Gilbert Teyangesikayi<br>David Fredericks	Groote Schuur Hospital
Ali Ait Hssain<br>Jeffrey Aliudin<br>Al-Reem Alqahtani<br>Khoulod Mohamed<br>Ahmed Mohamed<br>Darwin Tan<br>Joy Villanueva<br>Ahmed Zaqout<br>Ahmed Labib	Hamad General Hospital - Weill Cornell Medical College in Qatar

Ethan Kurtzman<br>Arben Ademi<br>Ana Dobrita<br>Khadija El Aoudi<br>Juliet Segura	Hartford HealthCare
Gezy Giwangkancana	Hasan Sadikin Hospital (Adult)
Shinichiro Ohshimo	Hiroshima University
Javier Osatnik	Hospital Alemán
Anne Joosten	Hospital Civil Marie Curie
Antoni Torres<br>Minlan Yang<br>Ana Motos	Hospital Clinic, Barcelona
Carlos Luna	Hospital de Clínicas
Francisco Arancibia	Hospital del Tórax
Virginie Williams<br>Alexandre Noel	Hospital du Sacre Coeur (Universite de Montreal)
Nestor Luque	Hospital Emergencia Ate Vitarte
Marina Fantini	Hospital Mater Dei
Ruth Noemi Jorge García<br>Enrique Chicote Alvarez	Hospital Nuestra Señora de Gracia
Anna Greti	Hospital Puerta de Hierro
Adrian Ceccato	Hospital Universitari Sagrat Cor
Angel Sanchez	Hospital Universitario Sant Joan d'Alacant
Ana Loza Vazquez	Hospital Universitario Virgen de Valme
Ferran Roche-Campo<br>Diego Franch-Llasat	Hospital Verge de la Cinta de Tortosa
Divina Tuazon	Houston Methodist Hospital
Marcelo Amato<br>Luciana Cassimiro<br>Flavio Pola<br>Francis Ribeiro<br>Guilherme Fonseca	INCOR (Universidade de São Paulo)
Heidi Dalton<br>Mehul Desai<br>Erik Osborn<br>Hala Deeb	INOVA Fairfax Hospital
Antonio Arcadipane<br>Gennaro Martucci<br>Giovanna Panarello<br>Stefano Vitiello<br>Claudia Bianco<br>Giovanna Occhipinti<br>Matteo Rossetti<br>Raffaele Cuffaro	ISMETT
Nidhal Siddig	Jabra Hospital, Khartoum
Sung-Min Cho<br>Glenn Whitman	Johns Hopkins

Marwan El Sayed<br>Walaa Mokhtar<br>Eslam El-Shenawy	Kafr Elsheikh University Hospital
Hiroaki Shimizu<br>Naoki Moriyama	Kakogawa Acute Care Medical Center
Jae-Burm Kim	Keimyung University Dong San Hospital
Nobuya Kitamura	Kimitsu Chuo Hospital
Johannes Gebauer	Klinikum Passau
Toshiki Yokoyama	Kouritu Tousei Hospital
Abdulrahman Al-Fares<br>Sarah Buabbas<br>Esam Alamad<br>Fatma Alawadhi<br>Kalthoum Alawadi<br>Mohamed Ahmed Khalefa<br>Nourah Ahmad Abdulaziz Al Ajeel<br>Mohammad Fathy Aly<br>Abdullah Al-Saleh<br>Abdullah Naanouh<br>Alaa Mohammed Elshourbgy<br>Abdulrahman Al-Fares<br>Mohamed Yousef Gad<br>Rania Mohamed ElRazaz<br>Ibrahim Khadadah<br>Ahmed Mohammed Almumin<br>Hala Altarakma<br>Hasan Albannay<br>Mohammed Kh Alsaleh<br>Mahmoud Saad Abdallah Radwan<br>Islam Ahmed Saadallah	Al-Amiri and Jaber Al-Ahmed Hospitals, Kuwait Extracorporeal Life Support Program
Hiro Tanaka	Kyoto Medical Centre
Satoru Hashimoto<br>Masaki Yamazaki	Kyoto Prefectural University of Medicine
Tak-Hyuck Oh	Kyung Pook National University Chilgok Hospital
Mark Epler<br>Cathleen Forney<br>Louise Kruse<br>Jared Feister<br>Joelle Williamson<br>Katherine Grobengieser	Lancaster General Health
Eric Gnall<br>Sasha Golden<br>Mara Caroline<br>Timothy Shapiro<br>Colleen Karaj<br>Lisa Thome<br>Lynn Sher<br>Mark Vanderland<br>Mary Welch<br>Sherry McDermott	Lankenau Institute of Medical Research (Main Line Health)

Matthew Brain<br>Sarah Mineall<br>Maria Unwin<br>Lixian Chen<br>Tarnya Trezise<br>Laurie McKeon	Launceston General Hospital
Dai Kimura	Le Bonheur Children's Hospital
Luca Brazzi<br>Gabriele Sales<br>Giorgia Montrucchio	Le Molinette Hospital (Ospedale Molinette Torino)
Tawnya Ogston	Legacy Emanuel Medical Center
Dave Nagpal<br>Karlee Fischer	London Health Sciences Centre
Roberto Lorusso<br>Bas van Bussell<br>Maria Elena De Piero<br>Silvia Mariani	Maastricht University Medical Centre
Dr Rajavardhan Rangappa<br>Dr Rajesh Mohan Shetty<br>Sujin Rai P<br>Argin Ganesan	Manipal Hospital Whitefield
Samar Tharwat	Mansoura University Hospital
Mariano Esperatti<br>Nora Angélica Fuentes<br>Maria Eugenia Gonzalez	Hospital Privado de Comunidad. Mar del Plata. Escuela Superior de Medicina. Universidad Nacional de Mar del Plata
Diarmuid O'Briain	Maroondah Hospital
Edmund G. Carton	Mater Misericordiae University Hospital
Ayan Sen<br>Amanda Palacios<br>Deborah Rainey	Mayo Clinic College of Medicine
Gordan Samoukoviv<br>Josie Campisi	McGill University Health Centre
Lucia Durham<br>Emily Neumann<br>Cassandra Seefeldt<br>Octavio Falcucci<br>Amanda Emmrich<br>Jennifer Guy<br>Carling Johns<br>Kelly Potzner<br>Catherine Zimmermann<br>Angelia Espinal	Medical College of Wisconsin (Froedtert Hospital)
Nina Buchtele<br>Michael Schwameis<br>Andrea Korhnfehl<br>Roman Brock<br>Thomas Staudinger	Medical University of Vienna
Stephanie-Susanne Stecher<br>Michaela Barnikel<br>Sófia Antón	Medical Department II, LMU Hospital Munich

Alexandra Pawlikowski	
Akram Zaaqoq<br>Lan Anh Galloway<br>Caitlin Merley	MedStar Washington Hospital Centre
Mohamed Muftah	Misurata Medical Center
Alistair Nichol	Monash University
Marc Csete<br>Luisa Quesada<br>Isabela Saba	Mount Sinai Medical Centre
Daisuke Kasugai<br>Hiroaki Hiraiwa<br>Taku Tanaka	Nagoya University Hospital
Eva Marwali<br>Yoel Purnama<br>Santi Rahayu Dewayanti<br>Ardiyan<br>Dafsah Arifa Juzar<br>Debby Siagian	National Cardiovascular Center Harapan Kita, Jakarta, Indonesia
Yih-Sharng Chen	National Taiwan University Hospital
Amer Aldhalia	Nasar City Hospital for Insurance
Mark Ogino	Nemours Alfred I duPont Hospital for Children
Prashant Nasa<br>Christina Matthew<br>Nimisha Abdul Majeed	NMC Al Nahda Hospital Dubai
Wael Hafez	NMC Royal Hospital, Abu Dhabi
Indrek Ratsep<br>Andra-Maris Post<br>Piret Sillaots<br>Anneli Krund<br>Merili-Helen Lehiste<br>Tanel Lepik	North Estonia Medical Centre
Frank Manetta<br>Effe Mihelis<br>Iam Claire Sarmiento<br>Mangala Narasimhan<br>Michael Varrone	Northwell Health
Mamoru Komats	Obihiro-Kosei General Hospital
Julia Garcia-Diaz<br>Catherine Harmon	Ochsner Clinic Foundation
S. Veena Satyapriya<br>Amar Bhatt<br>Nahush A. Mokadam<br>Alberto Uribe<br>Alicia Gonzalez<br>Haixia Shi<br>Johnny McKeown<br>Joshua Pasek<br>Juan Fiorda<br>Marco Echeverria	Ohio State University Medical Centre
Rita Moreno	Oklahoma Heart Institute

Bishoy Zakhary	Oregon Health and Science University Hospital (OHSU)
Marco Cavana<br>Alberto Cucino	Ospedale di Arco (Trento Hospital)
Giuseppe Foti<br>Marco Giani<br>Benedetta Fumagalli	Ospedale San Gerardo
Davide Chiumello<br>Valentina Castagna	Ospedale San Paolo
Andrea Dell'Amore<br>Paolo Navalesi	Padua University Hospital (Policlinico of Padova)
Hoi-Ping Shum	Pamela Youde Nethersole Eastern Hospital
Alain Vuysteke	Papworth Hospitals NHS Foundation Trust
Asad Usman<br>Andrew Acker<br>Benjamin Smood<br>Blake Mergler<br>Federico Sertic<br>Madhu Subramanian<br>Alexandra Sperry<br>Nicolas Rizer	Penn Medicine (Hospital of the University of Pennsylvania)
Erlina Burhan<br>Menaldi Rasmin<br>Ernita Akmal<br>Faya Sitompul<br>Navy Lolong<br>Bhat Naivedh	Persahabatan General Hospital
Simon Erickson	Perth Children's Hospital
Peter Barrett<br>David Dean<br>Julia Daugherty	Piedmont Atlanta Hospital
Antonio Loforte	Policlinico di S. Orsola, Università di Bologna
Irfan Khan<br>Mohammed Abraar Quraishi<br>Olivia DeSantis	Presbyterian Hospital Services, Albuquerque
Ahmad Nasrallah	Prince Hamza Hospital- Amman
Dominic So<br>Darshana Kandamby	Princess Margaret Hospital
Jose M. Mandei<br>Hans Natanael	Prof Dr R. D. Kandou General Hospital - Paediatric
Eka YudhaLantang<br>Anastasia Lantang	Prof Dr R. D R. D. Kandou General Hospital - Adult
Surya Oto Wijaya	Dr Sulianti Saroso Hospital
Anna Jung	Providence Saint John's Health Centre
George Ng<br>Wing Yiu Ng	Queen Elizabeth Hospital, Hong Kong
Pauline Yeung Ng<br>Shu Fang	The University of Hong Kong
Alexis Tabah<br>Megan Ratcliffe<br>Maree Duroux	Redcliffe Hospital

Ahmed Alajeeli<br>Ali Tarhabat	Regdalin Hospital
Shingo Adachi<br>Shota Nakao	Rinku General Medical Center (and Senshu Trauma and Critical Care Center)
Pablo Blanco<br>Ana Prieto<br>Jesús Sánchez	Rio Hortega University Hospital
Meghan Nicholson	Rochester General Hospital
Warwick Butt<br>Alyssa Serratore<br>Carmel Delzoppo	Royal Children's Hospital
Pierre Janin<br>Elizabeth Yarad	Royal North Shore Hospital
Richard Totaro<br>Jennifer Coles	Royal Prince Alfred Hospital
Bambang Pujo	RSUD Soetomo
Robert Balk<br>Andy Vissing<br>Esha Kapania<br>James Hays<br>Samuel Fox<br>Garrett Yantosh<br>Pavel Mishin	Rush University, Chicago
Safia Adem	Sabha Medical Center
Saptadi Yuliarto<br>Kohar Hari Santoso<br>Susanthy Djajalaksana	Saiful Anwar Malang Hospital (Brawijaya University) (Paediatrics)
Arie Zainul Fatoni	Saiful Anwar Malang Hospital (Brawijaya University) (Adult)
Masahiro Fukuda	Saiseikai Senri Hospital
Keibun Liu	Saiseikai Utsunomiya Hospital
Paolo Pelosi<br>Denise Battaglini<br>Chiara Robba	San Martino Hospital
Juan Fernando Masa Jiménez	San Pedro de Alcantara Hospital
Diego Bastos	Sao Camilo Cura D'ars
Sérgio Gaião	São João Hospital Centre, Porto
Desy Rusmawatiningtyas	Sardjito Hospital (Paediatrics)
Young-Jae Cho	Seoul National University Bundang Hospital
Su Hwan Lee	Severance Hospital
Tatsuya Kawasaki	Shizuoka Children's Hospital
Laveena Munshi	Sinai Health Systems (Mount Sinai Hospital)
Pranya Sakiyalak<br>Prompak Nitayavardhana	Siriraj Hospital
Mohamed Bashir Elagili<br>Talat Ahmed Abu Salem	Soug Althulatha Isolation Center
Tamara Seitz	Sozialmedizinisches Zentrum Süd – Kaiser-Franz-Josef-Spital
Rakesh Arora<br>David Kent	St Boniface Hospital (University of Mannitoba)
Daniel Marino	St Christopher's Hospital for Children

Swapnil Parwar<br>Andrew Cheng<br>Jennene Miller	St George Hospital
Shigeki Fujitani<br>Naoki Shimizu	St Marianna Medical University Hospital
Jai Madhok<br>Clark Owyang	Stanford University Hospital
Hergen Buscher<br>Claire Reynolds	St Vincent's Hospital
Abusalama Abdurraouf<br>Ali Abdulnasir Kredan<br>Abdurrahman Haddud	Swani Health Isolation Center
Saad Moharam	Tanta University Hospital
Olavi Maasikas<br>AleksanBeljantsev<br>Vladislav Mihnovits	Tartu University Hospital
Takako Akimoto<br>Mariko Aizawa<br>Kanako Horibe<br>Ryota Onodera	Teine Keijinkai Hospital
Carol Hodgson<br>Aidan Burrell<br>Meredith Young	The Alfred Hospital
Timothy George	The Heart Hospital Baylor Plano, Plano
Kiran Shekar<br>Niki McGuinness<br>Lacey Irvine	The Prince Charles Hospital
Brigid Flynn	The University of Kansas Medical Centre
Abdulrahman Almjersah	Tishreen University Hospital
Tomoyuki Endo	Tohoku Medical and Pharmaceutical University
Kazuhiro Sugiyama	Tokyo Metropolitan Bokutoh Hospital
Keiki Shimizu	Tokyo Metropolitan Medical Center
Eddy Fan<br>Kathleen Exconde	Toronto General Hospital
Shingo Ichiba	Tokyo Women's Medical University Hospital
Muhannud Binnawara	Tripoli Central Hospital
Hussein Embarek	Tripoli University Hospital
Leslie Lussier	Tufts Medical Centre (and Floating Hospital for Children)
Gösta Lotz	Universitätsklinikum Frankfurt (University Hospital Frankfurt) (Uniklinik)
Maximilian Malfertheiner<br>Lars Maier<br>Esther Dreier	Universitätsklinikum Regensburg (Klinik für Innere Medizin II)
Neurinda Permata Kusumastuti	University Airlangga Hospital (Paediatric)
Colin McCloskey<br>Al-Awwab Dabaliz<br>Tarek B Elshazly<br>Josiah Smith	University Hospital Cleveland Medical Centre (UH Cleveland Hospital)

Konstanty S. Szuldrzynski<br>Piotr Bielański	University Hospital in Krakow
Yusuff Hakeem	University Hospitals of Leicester NHS Trust (Glenfield Hospital)
Keith Wille	University of Alabama at Birmingham Hospital (UAB)
Srinivas Murthy	University of British Columbia
Ken Kuljit S. Parhar<br>Kirsten M. Fiest<br>Cassidy Codan<br>Anmol Shahid	University of Calgary (Peter Lougheed Centre, Foothills Medical Centre, South Health Campus and Rockyview General Hospital)
Mohamed Fayed<br>Timothy Evans<br>Rebekah Garcia<br>Ashley Gutierrez<br>Hiroaki Shimizu	University of California, San Francisco-Fresno Clinical Research Centre
Tae Song<br>Rebecca Rose	University of Chicago
Suzanne Bennett<br>Denise Richardson	University of Cincinnati Medical Centre
Giles Peek	University of Florida
Lovkesh Arora<br>Kristina Rappapport<br>Kristina Rudolph<br>Zita Sibenaller<br>Lori Stout<br>Alicia Walter	University of Iowa
Daniel Herr<br>Nazli Vedadi	University of Maryland - Baltimore
Robert Bartlett	University of Michigan Medical Center
Antonio Pesenti	University of Milan
Shaun Thompson<br>Julie Hoffman<br>Xiaonan Ying<br>Bailey Williams<br>Emely Sanchez<br>Chika Akwani	University of Nebraska Medical Centre
Ryan Kennedy	University of Oklahoma Health Sciences Centre (OU)
Muhammed Elhadi	Faculty of Medicine, University of Tripoli
Matthew Griffee<br>Mary Mone<br>Anna Ciullo<br>Yuri Kida	University of Utah Hospital
Ricard Ferrer Roca<br>JordI Riera<br>Sofia Contreras<br>Cynthia Alegre	Vall d'Hebron University Hospital, Barcelona
Christy Kay<br>Irene Fischer<br>Elizabeth Renner	Washington University in St. Louis/ Barnes Jewish Hospital

Hayato Taniguci	Yokohama City University Medical Center
James Lee<br>Daniel Plotkin<br>Barbara Wanjiru Citarella<br>Laura Merson	ISARIC, Centre for Tropical Medicine and Global Health, University of Oxford, Oxford, UK

Collaborators

Prefix/First Name/Last Name	Site Name
Emma Hartley	Aberdeen Royal Infirmary (Foresterhill Health Campus)
Bastian Lubis	Adam Malik Hospital
Takanari Ikeyama	Aichi Childrens Health and Medical Center
Alshaymaa Mortada	Ain Shams University
Ameen Alhamad	Aleppo University Hospital
Ahmed Mechi	Al-Sader Teaching Hospital, Al-Najaf
Islam Mohsen Ali Mohamed Hassan Nadar	Al Salam Specialized Hospital
Mohammed Saleh Alyasiri	Alshifa Center Medical City
Muhammed Zainab Alghali Elsaid	Alshuhada Hospital
Balu Bhaskar	American Hospital
Jae-Seung Jung	Anam Korea University Hospital
Shay McGuinness	Auckland City Hospital
Glenn Eastwood	Austin Hospital
Sandra Rossi Marta<br>Fabio Guarracino	Azienda Ospedaliero Universitaria Parma
Stacy Gerle	Banner University Medical Centre
Emily Coxon	Baptist Health Louisville
Bruno Claro	Barts Hospital
Wafa Aldressi	Benghazi Medical Centre
Mahmoud Eleisawy	Benha University Hospital
Hasnaa Osama	Beni-Suef University Hospital
Daniel Loverde	Billings Clinic
Namrata Patil	Brigham and Women’s Hospital
Vieri Parrini	Borgo San Lorenzo Hospital
Angela McBride	Brighton and Sussex Medical School
Kathryn Negaard	Brooke Army Medical Centre
Angela Ratsch	Bundaberg Hospital
Ahmad Abdelaziz	Cairo University Hospital
Juan David Uribe	Cardio VID
Adriano Peris	Careggi Hospital
Mark Sanders	Cedar Park Regional Medical Center
Dominic Emerson	Cedars-Sinai Medical Centre
Muhammad Kamal	Cengkareng Hospital
Hamza Faida	Centre Hospitalier Universitaire Ibn Sina Rabat

Pedro Povoa	Centro Hospitalar de Lisboa
Roland Francis	Charite-Univerrsitatsmedizi n Berlin
Ali Cherif	Charles Nicolle University Hospital
Sunimol Joseph	Children’s Health Ireland (CHI) at Crumlin
Matteo Di Nardo	Children’s Hospital Bambino Gesù
Micheal Heard	Children's Healthcare of Atlanta – Egleston Hospital
Kimberly Kyle	Children's Hospital – Los Angeles
Ray A Blackwell	Christiana Care Health System's Centre for Heart and Vascular Health
Amel OUYAHIA	CHU - Chu Saadna Abdenour De Sétif - Sétif
Michael Piagnerelli<br>Patrick Biston	CHU de Charleroi
Hye Won Jeong	Chungbuk National University Hospital
Reanna Smith	Cincinnati Children's
Yogi Prawira	Cipto Mangunkusumo Hospital
Giorgia Montrucchio	Città della Salute e della Scienza Hospital – Turin, Italy
Arturo Huerta Garcia	Clínica Sagrada Família
Nahikari Salterain	Clinica Universidad de Navarra
Bart Meyns	Collaborative Centre Department Cardiac Surgery, UZ Leuven
Muhammed Elnasser	Damascus Hospital
Marsha Moreno	Dignity Health Medical Group- Dominican
Rajat Walia	Dignity Health St. Joseph's Hospital and Medical Center (SJHMC)
Amit Mehta	Doernbecher Children’s Hospital
Annette Schweda	Donaustauf Hospital
Melissa Williams	Duke University Hospital (Durham)
Emad Amkhatirah	Elmarj Teaching Hospital
Kyung Hoon Kim	Eunpyeung St Mary's Hospital
Alexandra Assad	Fluminense Federal University
Estefania Giraldo	Fundación Clinica Shaio (Shaio Clinic)
Wojtek Karolak	Gdansk Medical University
Martin Balik	General University Hospital
Elizabeth Pocock	George Washington University Hospital
Akram Mohamed	Gharyan Central Hospital
Evan Gajkowski	Giesinger Medical Centre
Mohamed Bedair	Giza International Hospital
Kanamoto Masafumi	Gunma University Graduate School of Medicine
Nicholas Barrett	Guy's and St Thomas NHS Foundation Trust Hospital
Yoshihiro Takeyama	Hakodate City Hospital
Sunghoon Park	Hallym University Sacred Heart Hospital
Faizan Amin	Hamilton General Hospital
Fina Meilyana Andriyani	Hasan Sadikin Hospital (Paediatric)
Serhii Sudakevych	Heart Institute Ministry of Health of Ukraine
Janos Schnur	Heim Pál National Pediatric Institute
Angela Ratsch	Hervey Bay Hospital

Magdalena Vera	Hospital Clinico de la Pontificia Universidad Catolica
Rodrigo Cornejo	Hospital Clinico de la Universidad de Chile
Patrícia Schwarz<br>Ana Carolina Mardini	Hospital de Clínicas de Porto Alegre
Thais de Paula	Hospital Felicio Rocho
Ary Serpa Neto	Hospital Israelita Albert Einstein
Andrea Villoldo	Hospital Privado de Comunidad
Alexandre Siciliano Colafranceschi	Hospital Pro Cardíaco
Alejandro Ubeda Iglesias	Hospital Punta de Europa
Juan Granjean	Hospital Regional de Valdivia
Lívia Maria Garcia Melro<br>Giovana Fioravante Romualdo	Hospital Samaritano Paulista
Diego Gaia	Hospital Santa Catarina
Helmgton Souza	Hospital Santa Marta
Filomena Galas	Hospital Sirio Libanes
Rafael Máñez Mendiluce	Hospital Universitario de Bellvitge
Alejandra Sosa	Hospital Universitario Esperanza (Universidad Francisco Marroquin)
Ignacio Martinez	Hospital Universitario Lucus Augusti
Hiroshi Kurosawa	Hyogo Prefectural Kobe Children's Hospital
Mohammad Badr Almoshantaf	Ibn Al-Nafees Hospital
Juan Salgado	Indiana University Health
Beate Hugi-Mayr	Inselspital University Hospital
Eric Charbonneau	Institut Universitaire de Cardiologie et de Pneumologie de Quebec - Universite Laval
Vitor Salvatore Barzilai	Instituto de Cardiologia do Distrito Federal - ICDF
Veronica Monteiro	Instituto de Medicina Integral . Fernando Figueira (IMIP)
Rodrigo Ribeiro de Souza	Instituto Goiano de Diagnostico Cardiovascular (IGDC)
Michael Harper	INTEGRIS Baptist Medical Center
Hiroyuki Suzuki	Japan Red Cross Maebashi Hospital
Celina Adams	John C Lincoln Medical Centre
Jorge Brieva	John Hunter Hospital
Almu'atasim Khamees	Jordan University Hospital
Fadi Graige	Kalamoon Hospital
Moh Supriatna	Kariadi Hospital Semarang
George Nyale	Kenyatta National Hospital (KNH)
Faisal Saleem Eltatar	King Abdullah Medical City
Jihan Fatani	King Abdullah Medical City Specialist Hospital
Husam Baeissa	King Abdullah Medical Complex
Ayman AL Masri	King Salman Hospital NWAF
Ahmed Rabie	King Saud Medical City
Mok Yee Hui	KK Women's and Children's Hospital
Masahiro Yamane	KKR Medical Center
Hanna Jung	Kyung Pook National University Hospital
Ayorinde Mojisola Margaret	Lagos University Teaching Hospital

Newell Nacpil	Lung Center of the Philippines
Katja Ruck	Luxembourg Heart Center
Rhonda Bakken	M Health Fairview
Claire Jara	Maine Medical Centre (Portland Maine)
Tim Felton	Manchester University NHS Foundation Trust - Wythenshawe
Lorenzo Berra	Massachusetts General Hospital
Bobby Shah	Medanta Hospital
Arpan Chakraborty	Medica Super speciality Hospital
Monika Cardona	Medical University of South Carolina
Gerry Capatos	Mediclinic Parkview Hospital Dubai
Bindu Akkanti	Memorial Hermann - Texas Medical Centre
Abiodun Orija	Memorial Regional Hospital (Hollywood Florida)
Harsh Jain	Mercy Hospital of Buffalo
Asami Ito	Mie University Hospital
Brahim Housni	Mohammed VI University Hospital
Sennen Low	National Centre for Infectious Diseases
Koji Iihara	National Cerebral and Cardiovascular Center
Joselito Chavez	National Kidney and Transplant Institute
Kollengode Ramanathan	National University Hospital, Singapore
Gustavo Zabert	National University of Comahue
Krubin Naidoo	Nelson Mandela Children's Hospital
Ian Seppelt	Nepean Hospital
Marlice VanDyk<br>Sarah MacDonald	Netcare Unitas ECMO Centre
Shingo Ichiba	Nippon Medical School Hospital
Randy McGregor	Northwestern Medicine
Teka Siebenaler	Norton Children's Hospital
Hannah Flynn	Novant Health (NH) Presbyterian Medical Centre
Kristi Lofton	Ochsner LSA Health Shreveport
Toshiyuki Aokage	Okayama University Hospital
Bakar Kvirkvelia	Open Heart 5th Clinical Hospital, Tbilisi
Kazuaki Shigemitsu	Osaka City General Hospital
Andrea Moscatelli	Ospedale Gaslini
Giuseppe Fiorentino	Ospedali dei Colli
Matthias Baumgaertel	Paracelsus Medical University Nuremberg
Serge Eddy Mba	Parirenyatwa General Hospital
Jana Assy	Pediatric and Neonatal Cardiac Intensive Care at the American University
Amelya Hutahaean	Pelni Hospital
Holly Roush	Penn State Heath S. Hershey Medical Centre
Kay A Sichting	Peyton Manning Children's Hospital
Francesco Alessandri	Policlinico Umberto, Sapienza University of Rome
Debra Burns	Presbyterian Hospital, New York/ Weill Cornell Medical Centre
Taha Husayn Alkhubouli	Preventive Medicine Hospital

Ahmad Nasrallah	Prince Hamza Hospital- Amman
Ahmed Rabie	Prince Mohammed bin Abdulaziz Hospital
Gavin Salt	Prince of Wales
Carl P. Garabedian	Providence Sacred Heart Children's Hospital
Jonathan Millar<br>Malcolm Sim	Queen Elizabeth II University Hospital
Adrian Mattke	Queensland Children's Hospital
Danny McAuley	Queens University of Belfast
Jawad Tadili	Rabat University Hospital
Tim Frenzel	Radboud University Medical Centre
Amro Abuleil	Rafidia Surgical Hospital
Yaron Bar-Lavie	Rambam Hospital
Aaron Blandino Ortiz	Ramón y Cajal University Hospital
Jackie Stone	Rapha Medical Centre
Alexis Tabah	Redcliffe Hospital
Antony Attokaran	Rockhampton Hospital
Michael Farquharson	Royal Adelaide Hospital
Brij Patel	Royal Brompton & Harefield NHS Foundation Trust
Derek Gunning	Royal Columbian Hospital
Kenneth Baillie	Royal Infirmary Edinburgh
Pia Watson	Sahlgrenska University Hospital
Kenji Tamai	Saiseikai Yokohamashi Tobu Hospital
Gede Ketut Sajinadiyasa<br>Dyah Kanyawati	Sanglah General Hospital
Marcello Salgado	Santa Casa de Misericordia de Juiz de Fora
Assad Sassine	Santa Casa de Misericórdia de Vitoria
Bhirowo Yudo	Sardjito Hospital
Scott McCaul	Scripps Memorial Hospital La Jolla
Bongjin Lee	Seoul National University Children's Hospital
Sang Min Lee	Seoul National University Hospital
Arnon Afek	Sheba Medical Center
Shimaa E Fattouh	Sherbin General Hospital
Yoshiaki Iwashita	Shimane University Hospital
Hammad Fadlalmola	Soba University Hospital
Bambang Pujo Semedi<br>Neurinda Permata Kusumastuti	Soetomo General Hospital (FK UNAIR)
Noureldin Mohamed Mansour	Souad Kafafi University Hospital
Jack Metiva	Spectrum Health Western Governors University
Nicole Van Belle	St. Antonius Hospital
Ignacio Martin-Loeches	St James's University Hospital
Dr Mohammed Al-Sadawi	Stony Brook University
Cenk Kirakli	Suat Seren Chest Diseases and Surgery Practice and Training Centre
Al-Touny Shimaa	Suez Canal University Hospitals
Lenny Ivatt	Swansea Hospital

Chia Yew Woon	Tan Tock Seng Hospital
Hyun Mi Kang	The Catholic University of Seoul St Mary Hospital
Timothy Smith	The Christ Hospital
Erskine James	The Medical Centre Navicent Health
Nawar Al-Rawas	Thomas Jefferson University Hospital
Yudai Iwasaki	Tohoku University
Hamza Ashour	Traghen Hospital
Kenny Chan King-Chung	Tuen Mun Hospital
Vadim Gudzenko	UCLA Medical Centre (Ronald Regan)
Beate Hugi-Mayr	Universitätsspital Bern, Universitätsklinik für Herz- und Gefässchirurgie
Fabio Taccone	Universite Libre de Bruxelles
Fajar Perdhana	University Airlangga Hospital (Adult)
Yoan Lamarche	University de Montreal (Montreal Heart Institute)
Joao Miguel Ribeiro	University Hospital CHLN
Nikola Bradic	University Hospital Dubrava
Klaartje Van den Bossche	University Hospital Leuven
Oude Lansink	University Medical Center Groningen
Gurmeet Singh	University of Aberta (Mazankowski Heart Institute)
Gerdy Debeuckelaere	University of Antwerp
Henry T. Stelfox	University of Calgary and Alberta Health Services
Cassia Yi	University of California at San Diego
Jennifer Elia	University of California, Irvine
Thomas Tribble	University of Kentucky Medical Center
Shyam Shankar	University of Missouri
Raj Padmanabhan	University of Pittsburgh Medical Centre
Bill Hallinan	University of Rochester Medical Centre (UR Medicine)
Luca Paoletti	University of South Carolina
Yolanda Leyva	University of Texas Medical Branch
Tatuma Fykuda	University of the Ryukyus
Jenelle Badulak	University of Washington in Seattle
Jillian Koch	University of Wisconsin & American Family Children's Hospital
Lisa Janowaik	UTHealth (University of Texas)
Amy Hackman	UT Southwestern
Deb Hernandez	Valley Children's Hospital (Madera)
Jennifer Osofsky	Vassar Brothers Medical Center (VBMC)
Katia Donadello	Verona Integrated University Hospital
Aizah Lawang	Wahidin Sudirohusodo Hospital
Josh Fine	WellSpan Health - York Hospital
Benjamin Davidson	Westmead Hospital
Andres Oswaldo Razo Vazquez	Yale New Haven Hospital
Ibrahim Abdehaleem	Zagazig University Hospital

Table S2. Normality testing for tabulated variables using Kolmogovor-Smirnov (K.S) testing

	K.S test statistic	P-Value
Age (years)	0.06	<0.01
BMI (kg/m^2)	0.11	<0.01
SOFA score	0.16	<0.01
SpO2 (%)	0.48	<0.01
Respiratory rate (breaths/min)	0.11	<0.01
Length of hospital stay (days)	0.17	<0.01
Nasal Flow	0.16	<0.01
MV Flow	0.11	<0.01
Nasal Duration	0.16	<0.01
MV Duration	0.21	<0.01
